# Potential impact of blood cholesterol guidelines on statin treatment in the U.S. population using interrupted time series analysis

**DOI:** 10.1186/s12872-024-03921-z

**Published:** 2024-05-10

**Authors:** Pui Ying Yew, Matt Loth, Terrence J. Adam, Julian Wolfson, Yue Liang, Peter J. Tonellato, Chih-Lin Chi

**Affiliations:** 1 Institute for Health Informatics, University of Minnesota, Minneapolis, MN USA; 2 School of Nursing, University of Minnesota, Minneapolis, MN USA; 3 Department of Pharmaceutical Care & Health Systems, University of Minnesota, Minneapolis, MN USA; 4 Division of Biostatistics, University of Minnesota, Minneapolis, MN USA; 5 Department of Health Management and Informatics, University of Missouri School of Medicine, Columbia, MO USA; 6 University of Minnesota Institute for Health Informatics, 8-102 Phillips-Wangensteen Building 516 Delaware St. SE, Minneapolis, MN 55455 USA

**Keywords:** MACE, Long-term effect, Guideline implementation, Policy effect, Generic atorvastatin, Interrupted time series analysis, Cardiovascular disease, Diabetes

## Abstract

**Background:**

The *2013 ACC/AHA Guideline* was a paradigm shift in lipid management and identified the four statin-benefit groups. Many have studied the guideline’s potential impact, but few have investigated its potential long-term impact on MACE. Furthermore, most studies also ignored the confounding effect from the earlier release of generic atorvastatin in Dec 2011.

**Methods:**

To evaluate the potential (long-term) impact of the *2013 ACC/AHA Guideline* release in Nov 2013 in the U.S., we investigated the association of the *2013 ACC/AHA Guideline* with the trend changes in 5-Year MACE survival and three other statin-related outcomes (statin use, optimal statin use, and statin adherence) while controlling for generic atorvastatin availability using interrupted time series analysis, called the Chow’s test. Specifically, we conducted a retrospective study using U.S. nationwide de-identified claims and electronic health records from Optum Labs Database Warehouse (OLDW) to follow the trends of 5-Year MACE survival and statin-related outcomes among four statin-benefit groups that were identified in the *2013 ACC/AHA Guideline*. Then, Chow’s test was used to discern trend changes between generic atorvastatin availability and guideline potential impact.

**Results:**

197,021 patients were included (ASCVD: 19,060; High-LDL: 33,907; Diabetes: 138,159; High-ASCVD-Risk: 5,895). After the guideline release, the long-term trend (slope) of 5-Year MACE Survival for the Diabetes group improved significantly (*P* = 0.002). Optimal statin use for the ASCVD group also showed immediate improvement (intercept) and long-term positive changes (slope) after the release (*P* < 0.001). Statin uses did not have significant trend changes and statin adherence remained unchanged in all statin-benefit groups. Although no other statistically significant trend changes were found, overall positive trend change or no changes were observed after the *2013 ACC/AHA Guideline* release.

**Conclusions:**

The *2013 ACA/AHA Guideline* release is associated with trend improvements in the long-term MACE Survival for Diabetes group and optimal statin use for ASCVD group. These significant associations might indicate a potential positive long-term impact of the *2013 ACA/AHA Guideline* on better health outcomes for primary prevention groups and an immediate potential impact on statin prescribing behaviors in higher-at-risk groups. However, further investigation is required to confirm the causal effect of the *2013 ACA/AHA Guideline.*

**Supplementary Information:**

The online version contains supplementary material available at 10.1186/s12872-024-03921-z.

## Background

Statins, also known as HMG-CoA reductase inhibitors, have been widely used to treat atherosclerotic cardiovascular disease (ASCVD), the leading cause of death in the United States (U.S.). Numerous randomized clinical trials have proven the efficacy of statins in lowering low-density lipoprotein cholesterol (LDL-C) and its association with a 20–30% reduction in death and major adverse cardiac events (MACE) per 1 mmol/L (39 mg/dL) reduction of LDL-C [1]. In Nov 2013, the American College of Cardiology (ACC) / American Heart Association (AHA) incorporated results from these clinical trials and released the 2013 ACC/AHA Guideline on the Treatment of Blood Cholesterol to Reduce Atherosclerotic Cardiovascular Risk in Adults [2] (which we refer to as the ‘*2013 ACC/AHA Guideline*’). The *2013 ACC/AHA Guideline* expanded recommendations to older cohorts and shifted from a “treat-to-LDL-target” approach in the previous guideline [3] to a focus on higher-intensity statins to reduce ASCVD risk using proven interventions. Given its simple structures and the attention that it received, many considered the *2013 ACC/AHA Guideline* successful, and it was built onto the recent 2018 guideline with additional recommendations for ezetimibe and PCSK9 inhibitors [4].

Following the *2013 ACC/AHA Guideline* release, some studies projected the uptake of higher-intensity statins among the statin-benefit groups, as well as the MACE prevention and treatment cost reduction [5–7]. While many studies followed up on these initial projections by conducting retrospective studies to analyze the association of the *2013 ACC/AHA Guideline* with statin-related outcomes [8–11], its association with long-term impact on MACE has yet to be investigated. It has been 10 years since the *2013 ACC/AHA Guideline* was first released and with the rise of availability of large observational data, it presented a unique opportunity to finally understand the trend of long-term MACE after the *2013 ACC/AHA Guideline* release. Understanding its potential long-lasting impact on MACE is crucial in managing public health because healthcare professionals can identify subgroups who can get better health outcomes (i.e., avoid MACE) and target subgroups who can benefit from more aggressive statin treatment strategies. Although there may not be enough data to understand the newer guideline (2018 guideline), since the *2013 ACC/AHA Guideline* is the pillar of the newer guideline, understanding the former can also provide healthcare professionals some insights into the latter.

Furthermore, among studies that have investigated the *2013 ACC/AHA Guideline* retrospectively, most only compared outcomes at two time points before and after the *2013 ACC/AHA Guideline*. However, Markovitz et al. [12] noted a potentially key confounding factor complicating the analysis of the potential impact of the *2013 ACC/AHA Guideline*: increased access to new generic atorvastatin. In Jun 2011, a potent statin, simvastatin 80 mg was restricted by the U.S. Food and Drug Administration (FDA) due to increased risk of muscle pain [13]. Five months later, another potent statin, atorvastatin, went off its patent and became available as a generic medication. Markovitz et al. [12] found that administrative policies such as the immediate access to a higher potency statin, atorvastatin, which replaced the recently restricted simvastatin 80 mg, had a greater effect on the increased adoption rate of high-intensity statins than did the *2013 ACC/AHA Guideline*. However, Markovitz et al.’s study and the small number of others who accounted for the accessibility to atorvastatin included only veterans or a small number of states within the U.S. [12, 14, 15], while we found that the Midwest region showed disparate trends in statin initiation and high-intensity atorvastatin adoption from the other U.S. regions (Supplemental Figure B-1). Despite the potentially limited generalizability, their results demonstrated the importance of isolating the sequential effects of these two policies: the addition of atorvastatin to the formulary and the release of the *2013 ACC/AHA Guideline*. Moreover, the usage of atorvastatin among the U.S. population increased several folds after atorvastatin became generic, shortly before the *2013 ACC/AHA Guideline* was released (Supplemental Figure B-2), so ignoring this factor could induce bias assessments of the potential impacts of guideline adoption on a variety of statin-related outcomes including statin use, optimal statin use, statin adherence, and MACE.

In this study, we aimed to apply a principled analytic approach to obtain national estimates of the potential impacts of the 2013 *ACC/AHA Guideline* on long-term MACE survival and other statin-related outcomes by controlling the confounding effect from generic atorvastatin availability on the four statin-benefit groups defined in the guideline: individuals with (1) ASCVD, (2) High-LDL, (3) Diabetes, and (4) High-ASCVD-Risk.

## Methods

### Data source

We conducted a retrospective study using U.S. nationwide de-identified longitudinal records from Optum Labs Database Warehouse (OLDW). The OLDW contains de-identified retrospective administrative claims and electronic health record (EHR) data on enrollees and patients, representing a mixture of ages and geographical regions across the U.S. The claims data in OLDW includes medical and pharmacy claims, laboratory results, and enrollment records for over 200 M commercial and Medicare Advantage enrollees. The EHR-derived data includes a subset of EHR data that has been normalized and standardized into a single database [16].

### Cohort selection

**Fig. 1:**
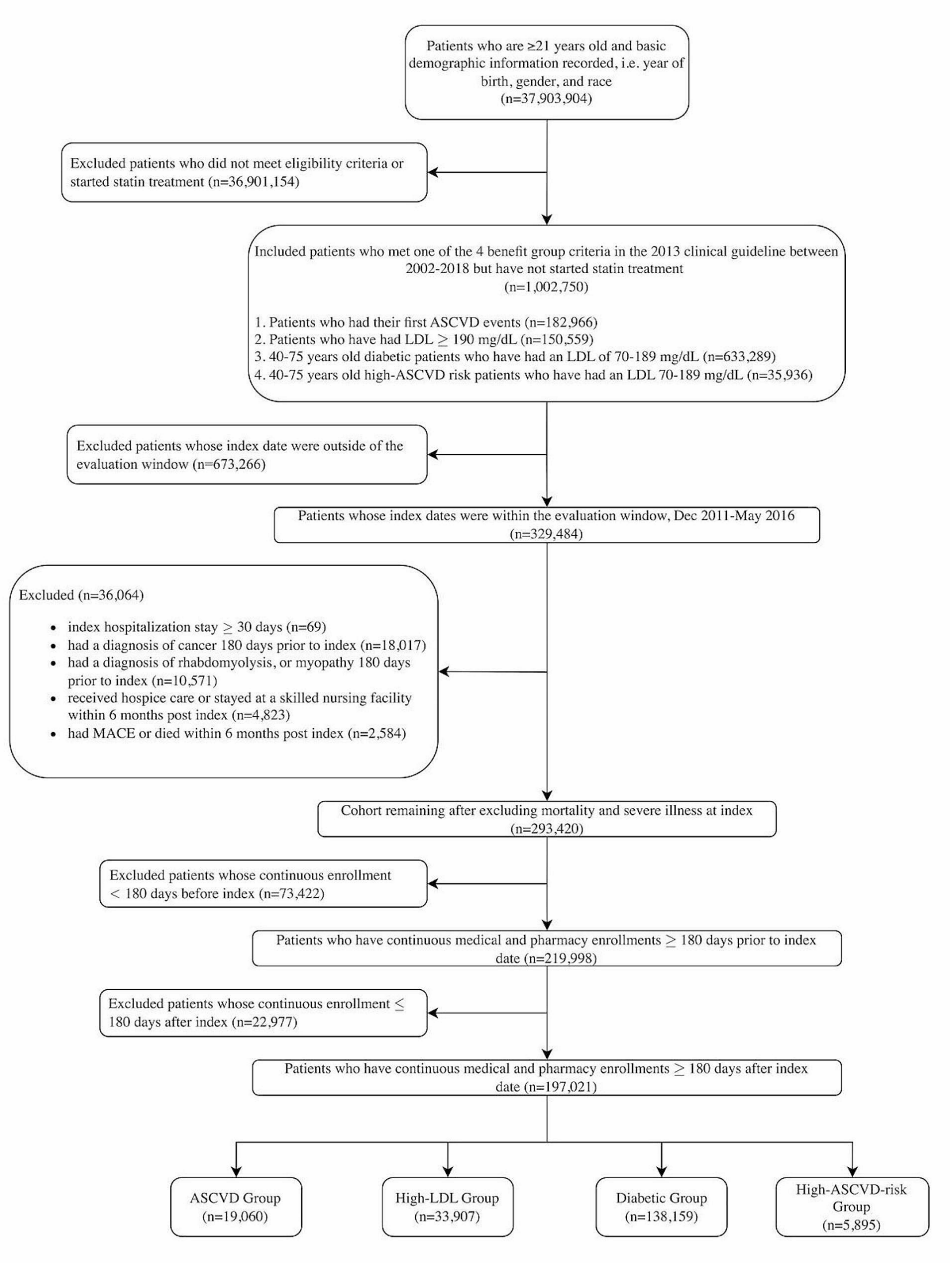
CONSORT Diagram. The flow of our study cohort selection process is shown in the figure above. A series of inclusion and exclusion criteria were applied and the resulting cohort description and size are reported

This study included patients aged 21 years old and above who belonged to one of the *2013 ACC/AHA Guideline* statin-benefit groups at a defined index (i.e., cohort entry) date within the evaluation window, which we defined as being between the first availability of generic atorvastatin in Dec 2011 to first availability of generic rosuvastatin in May 2016 (Fig. 1). The atorvastatin generic date was chosen as the start date of the evaluation window to avoid confounding effects from the availability of generic atorvastatin (i.e., all subjects’ follow-up starts after the availability of generic atorvastatin, so changes in outcomes are more plausibly attributable to the 2013 *ACC/AHA Guideline*), and the rosuvastatin generic date was chosen as the end of the evaluation window to avoid any confounding effect from the availability of generic rosuvastatin. Patients were assigned to one of the four statin-benefit groups that were identified by the *2013 ACC/AHA Guideline* [2], based on their clinical status at their index date: (1) the ASCVD group included patients who had clinical ASCVD; (2) the High-LDL group included patients who had LDL-C levels $\ge$ 190 mg/dL; (3) the Diabetes group included patients with diabetes who were aged 40–75 years old at cohort entry with LDL-C levels between 70 and 189 mg/dL; (4) the High-ASCVD-Risk group included patients who were aged 40–75 years old at cohort entry and had 10-year ASCVD risk $\ge$ 7.5% with LDL-C levels between 70 and 189 mg/dL. We used laboratory test results and vital status within 365 days of the index date to compute the patient’s 10-year ASCVD risk. The index date was defined as the date when the patient was first eligible to be considered for one of the statin-benefit groups, and cohort month was defined as the month of the index date. The four statin-benefit groups were mutually exclusive; in the event in which a patient was eligible to be in more than one statin-benefit group, only the first statin-eligible criteria and date were used. For example, if a patient had a high LDL-C value of 200 mg/dL in Jan 2012 and suffered a myocardial infarction in Dec 2013, the patient would be categorized as being in the High-LDL group in Jan 2012. Further description of cohort selection and detailed medical code phenotyping are available in the Supplemental Methods.

**Fig. 2:**
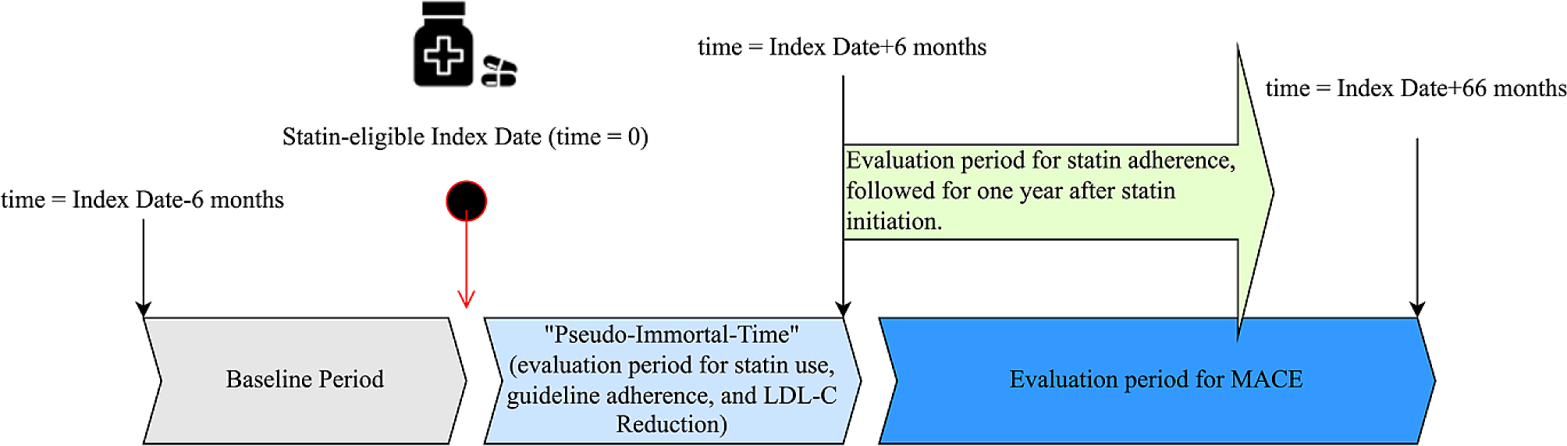
Timeline of our study. This figure describes the timeline of our study and the time of evaluation for each statin-related outcome

Further inclusion criteria included continuous medical and pharmacy insurance enrollment for six months before and after the index date to identify patient baseline characteristics and to capture statin use status within six months. Patients were required to have no history of statin use before the index date. Patients who met the criteria for any of the four statin-benefit groups before Dec 2011 were also excluded. This allowed the study to focus on the initial clinician-patient consultation in starting appropriate statin treatment. Further exclusion criteria included a diagnosis of cancer or serious muscle disorders, such as myopathy and rhabdomyolysis [17] at any time within six months before the index date. Due to the constraints we imposed to capture statin use status within six months of the index date, a six-month *pseudo-immortal-time* period was used in MACE analysis to avoid underestimation of severe MACE, i.e., deaths (Fig. 2) in this period. Patients who had an occurrence of MACE, death, hospice care, or skilled nursing facility admission during the *pseudo-immortal-time* period were excluded from this study.

### 2013 ACC/AHA guideline exposure

The exposure of this study is the *2013 ACC/AHA Guideline*. To control the effect from the generic atorvastatin availability, patients whose index dates were on or after Dec 2011 (atorvastatin generic date) but before Nov 2013 (*2013 ACC/AHA Guideline* release), were categorized as the “Pre-Guideline” (control) group. Patients whose index dates were on or after Nov 2013 but on or before May 2016 (rosuvastatin generic date), were categorized as the “Post-Guideline” (treatment) group.

### Statin-related outcomes

Four statin-related outcomes were analyzed in this study: (1) statin use six months after the index date, (2) optimal statin use among statin initiators, (3) statin adherence in one-year post initial statin use, and (4) MACE survival rate in five years after the *pseudo-immortal-time* period. Each of the four outcomes was assessed monthly, for each cohort month, for four statin-benefit groups respectively. A detailed definition of each statin-related outcome was described in Table 1.

**Table 1:** Statin-related outcome definitions

	Statin-Related Outcome	Definition in This Study
1.	Statin Use	Proportion of patients who received their initial statin prescription within six months after index date. Statin prescriptions were identified via pharmacy claims for lovastatin, simvastatin, pravastatin, fluvastatin, pitavastatin, atorvastatin, and rosuvastatin.
2.	Optimal Statin Use	Proportion of patients who were prescribed the optimal statin treatments among patients who initiated statin treatment in 6 months post index date. We defined optimal statin intensity as the recommended statin intensity by the *2013 ACC/AHA Guideline*^*a*^ (see Supplemental Figure A-1). Note: In the event that the *2013 ACC/AHA Guideline* recommended either moderate- or high-intensity, both prescription intensities were considered optimal statin treatments.
3.	Statin Adherence	Average proportion days covered (PDC)^b^ among patients who started their first statin treatment within one year following their index date. PDC was calculated by taking the sum of days supply of statin pharmacy prescriptions divided by the number of continuous enrollment days within one year after the statin initiation date.
4.	5-Year MACE^c^ Survival	Survival rate of MACE (cardiovascular or sudden death, non-fatal myocardial infarction, non-fatal stroke, hospitalization for unstable angina, or revascularization procedures) within five years of the end of the *pseudo-immortal-time* period. Kaplan-Meier estimate was fitted for each cohort month using the survival package in R v4.2.1 to adjust for potential loss to follow-up.

^a^*2013 ACC/AHA Guideline* = 2013 American College of Cardiology/American Heart Association Guideline on the Treatment of Blood Cholesterol to Reduce Atherosclerotic Cardiovascular Risk in Adults

^b^ PDC = Proportion Days Covered. This is calculated based on Nau DP (2012). Nau DP. Proportion of Days Covered (PDC) as a Preferred Method of Measuring Medication Adherence. *Pharm Qual Alliance*. 2012;6:3

^c^ MACE = Major Adverse Cardiac Events. 4-Point MACE is adopted based on the definition illustrated by Bosco et al. Bosco E, Hsueh L, McConeghy KW, Gravenstein S, Saade E. Major adverse cardiovascular event definitions used in observational analysis of administrative databases: a systematic review. *BMC Med Res Methodol*. 2021;21:241

^d^ Therneau TM. A Package for Survival Analysis in R. 2020

### Statistical analysis

As mentioned earlier, studies that only compare two time points before and after cannot account for the underlying trend, i.e., the confounding effect from atorvastatin went generic. To account for the confounding effect, we adopted interrupted time series analysis to identify the association of *2013 ACC/AHA Guideline* with potential (long-term) impacts on statin-related outcomes (i.e., abruptly changed the outcome trends). Interrupted time series analysis has been considered one of the stronger experimental designs when conducting a randomized clinical trial is not feasible because it tracks the outcome before and after longitudinally.

In this study, we adopted a commonly used interrupted time series analysis method, the Chow’s test, also sometimes referred to the segmented linear regression. The Chow’s test is used to detect significant trend changes in our outcomes at the time of the *2013 ACC/AHA Guideline* release and it models:

$$Y(t) = \left\{ {\begin{array}{*{20}{c}}{{\beta _{10}} + {\beta _{11}}(t),}&{t\,\varepsilon \,\left\{ {Dec\,2011,\,Oct\,2013} \right\}}\\{{\beta _{20}} + {\beta _{21}}(t),}&{t\,\varepsilon \,\left\{ {Nov\,2013,\,May\,2016} \right\}}\end{array}} \right.$$

where $Y\left(t\right)$ represents each of the statin-related outcomes (Table 1), ${\beta }_{10}$ and ${\beta }_{20}$ denote the intercepts of the linear regression lines while ${\beta }_{11}$ and ${\beta }_{21}$ denote the slopes of the linear regression lines. This model can detect both “immediate” changes in the mean of ${Y}_{t}$ by comparing the intercepts ${\beta }_{10}$ and ${\beta }_{20}$, as well as changes in the long-tern trends by comparing the slopes ${\beta }_{11}$ and ${\beta }_{21}$. Chow’s test can be used to test the null hypotheses that ${\beta }_{10}={\beta }_{20}$ and ${\beta }_{11}{=\beta }_{21}$, i.e., that the linear regression line is the same before (pre-guideline) and after (post-guideline) the *2013 ACC/AHA Guideline* release in Nov 2013. We applied Chow’s test using the strucchange package [18] in R v4.2.1. The threshold for significance was set at 0.05.

Aside from the *p*-values obtained from Chow’s test, the slopes of pre- and post-guideline periods were summarized in percentage points (pp) per year, i.e., ${\beta }_{11}$ and ${\beta }_{21}$(both represented monthly rates) multiplied by 12.

## Results

All results were summarized in the following group order unless specified: ASCVD, High-LDL, Diabetes, and High-ASCVD-Risk. A total of 197,021 patients were included in the evaluation window between Dec 2011 (atorvastatin generic date) and May 2016 (rosuvastatin generic date): 19,060 ASCVD (10%), 33,907 High-LDL (17%), 138,159 Diabetes (70%), 5,895 High-ASCVD-Risk (3%). The post-guideline sample size in the High-ASCVD-Risk group was more than three times the pre-guideline, while the average ratio of patients in the post- to pre-guideline periods was 1.4 for the other groups.

Table 2 summarizes patient characteristics at index and patient characteristics at the end of the *pseudo-immortal-time* period are described in Supplemental Table B-1. The patient distributions at index and 6 months after index did not change significantly. At index, the average age in the post-guideline was older than the pre-guideline for all statin-benefit groups (63.1–64.1; 50.9–51.4; 55.6–56.2; 59.5–61.3). The ratio of females to males was similar for all statin-benefit groups, aside from the High-ASCVD-Risk group having predominantly females (90-92%). The majority of the study population was white, especially in the ASCVD group (76%-76%). Contrastingly, the High-ASCVD-Risk group had a higher proportion of Black individuals (57%-51%). Age-adjusted Charlson Scores were higher in post-guideline in all statin-benefit groups but highest on average in the ASCVD group (4.00-4.28; 1.12–1.18; 2.21–2.42; 1.87–2.06). Notably, the ASCVD group had a higher prevalence of myocardial infarction, while all statin-benefit groups had a higher prevalence of obesity, diabetes with chronic complications, and tobacco use but lower ACE inhibitors use in post-guideline.

**Fig. 3:**
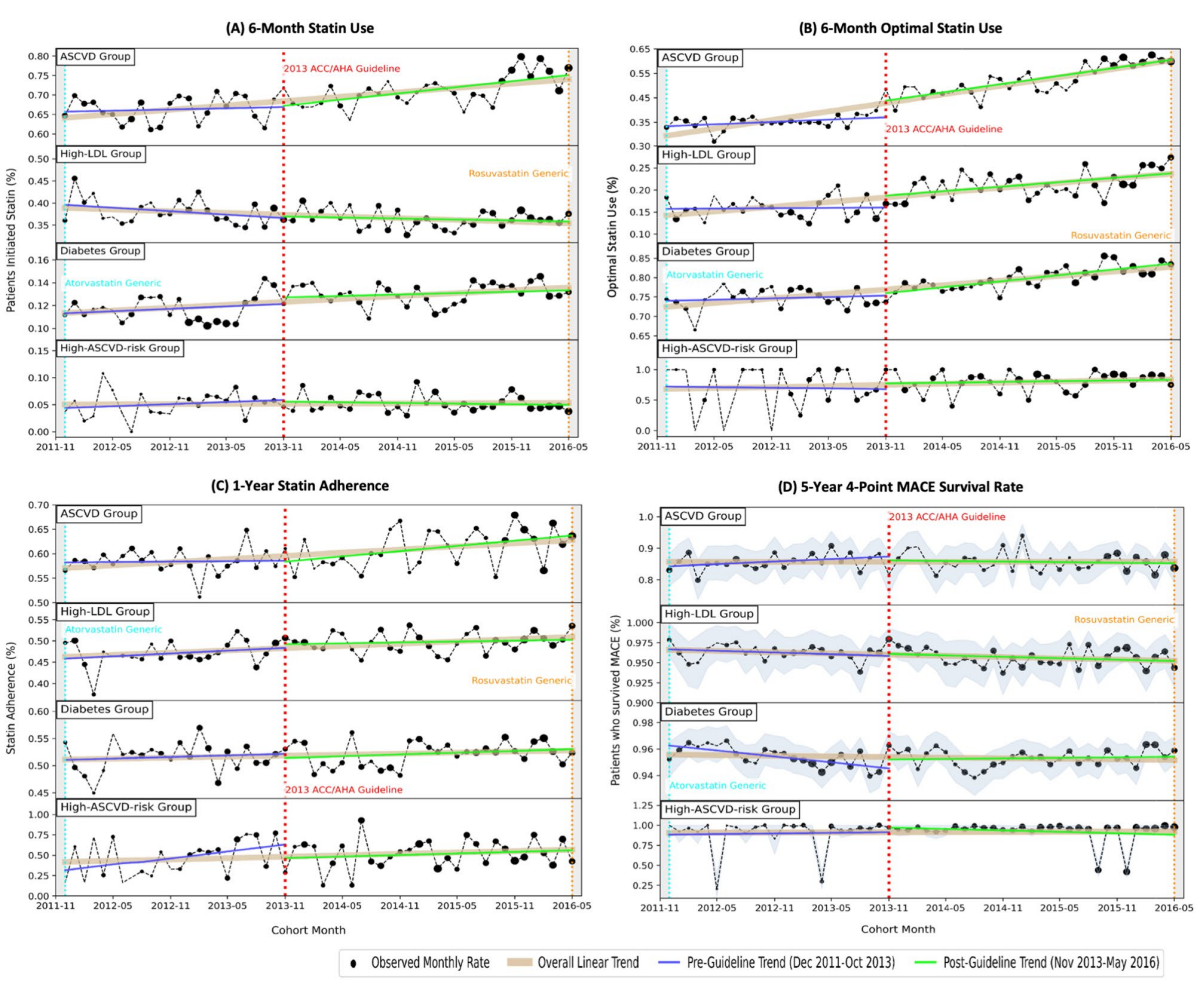
Statin-Related Outcome Trends. The figure shown above shows the monthly trends of the four statin-related outcomes from the atorvastatin generic date (Dec 2011) to the rosuvastatin generic date (May 2016) in four subplots: (**A**) 6-Month Statin Use, (**B**) 6-Month Optimal Statin Use, (**C**) 1-Year Statin Adherence, and (**D**) 5-Year MACE Survival. In each subplot, the outcome trends are shown separately for four statin-benefit groups on four different rows in the following order: ASCVD, High-LDL, Diabetes, and High-ASCVD-risk groups. A red vertical dotted line is drawn at the time of the *2013 ACC/AHA Guideline* release (Nov 2013) to distinguish the trends in the pre-guideline (Dec 2011-Oct 2013) and post-guideline period (Nov 2013-May 2016). The observed monthly outcome rates are plotted in a black circle marker according to the sample sizes of each month. The best-fitted lines resulting from linear regression models are shown in blue and green lines for pre- and post-guideline periods respectively, and in light brown shades for the entire study period (Dec 2011-May 2016). In subplot (**D**), the confidence intervals of the MACE survival rate estimated from the Kaplan-Meier curve are shaded in grey

**Fig. 4:**
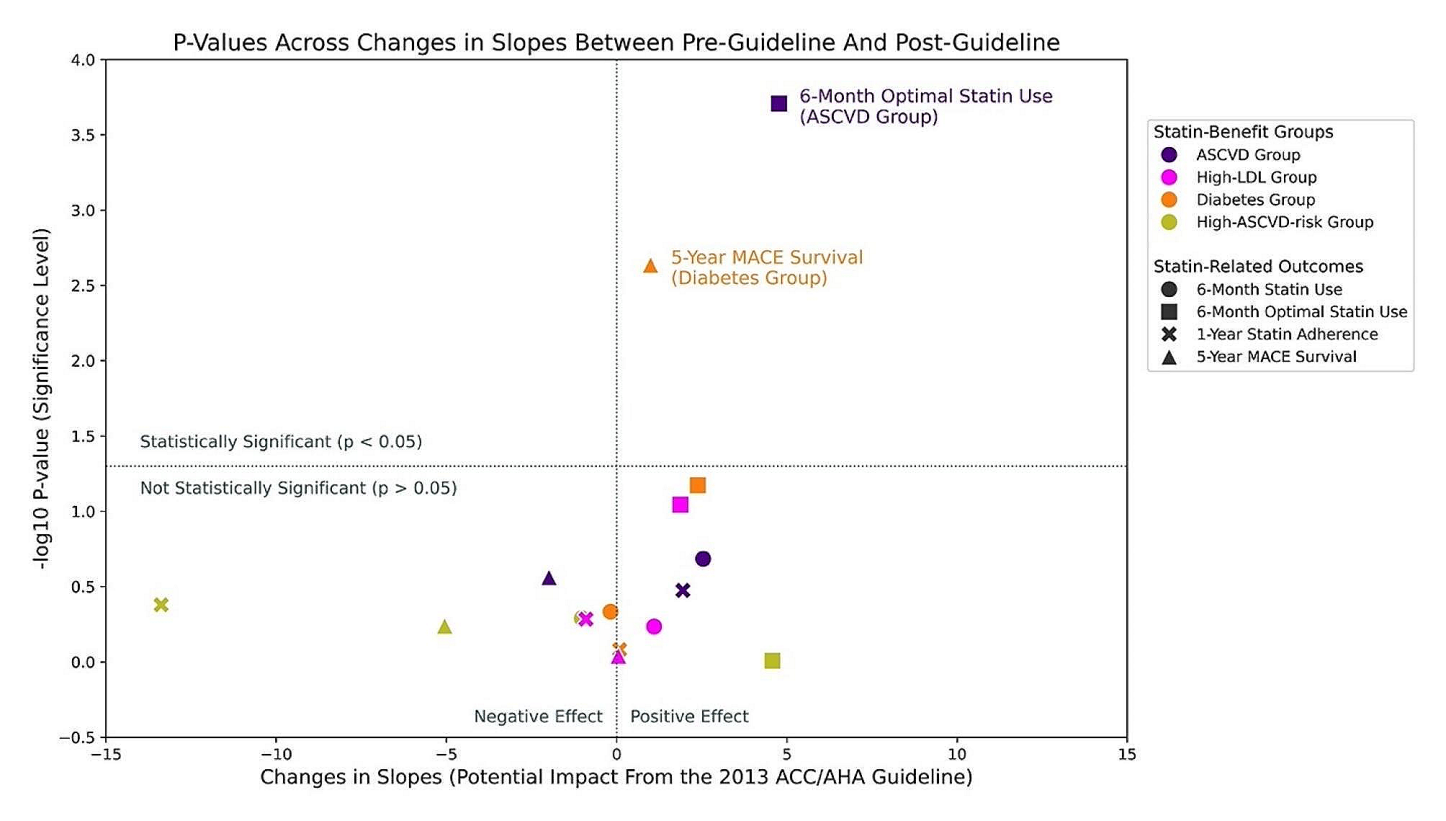
*P*-Values Across Changes in Slopes Between Pre-Guideline and Post-Guideline. This figure summarizes the findings of the Chow’s test, investigating if there is a significant trend changes associated with the the *2013 ACC/AHA Guideline* release in Nov 2013 in four statin-related outcomes (6-Month Statin Use, 6-Month Optimal Statin Use, 1-Year Statin Adherence, 5-Year MACE Survival) for each of the four statin-benefit groups (ASCVD, High-LDL, Diabetes, and High-ASCVD-risk groups). The four statin-related outcomes are drawn in different shapes of markers while the four statin-benefit groups are drawn in different colors. The changes in slopes (long-term trend changes) between pre-guideline and post-guideline are represented in the x-axis and their statistical significance (*p*-values from Chow’s test) are represented in the y-axis in a logarithmic scale. The vertical dotted line at 0 represents no changes in the slopes while the horizontal dotted line represents the statistical significance level from Chow’s test. Points on the top right corner are statistically significant positive changes in slopes while points on top left corner are statistically significant negative changes in slopes

Figure  3 illustrates the four statin-related outcome trends after atorvastatin went generic in Dec 2011 (pre-guideline) and trends after the *2013 ACC/AHA Guideline* release in Nov 2013 (post-guideline). Figure 4 summarizes the findings of the Chow’s test. In the following section, we discussed statin-related outcomes from pre- to post-guideline in the following order: statin use, optimal statin use, statin adherence, and MACE survival.

**Table 2:** Patient characteristics at index

	ASCVD^a^		High-LDL		Diabetes		High-ASCVD-Risk	
	Pre-Guideline (*n* = 8,290)	Post-Guideline (*n* = 10,770)	Pre-Guideline (*n* = 13,081)	Post-Guideline (*n* = 20,826)	Pre-Guideline (*n* = 59,219)	Post-Guideline (*n* = 78,940)	Pre-Guideline (*n* = 1,446)	Post-Guideline (*n* = 4,449)
Age	63.10 ± 12.71	64.10 ± 13.41	50.95 ± 12.47	51.45 ± 12.85	55.67 ± 9.71	56.20 ± 9.94	59.51 ± 10.77	61.35 ± 10.56
Ages 21–44	724 (0.09)	949 (0.09)	4,049 (0.31)	6,218 (0.30)	9,293 (0.16)	12,065 (0.15)	170 (0.12)	404 (0.09)
Ages 45–69	4,585 (0.55)	5,616 (0.52)	8,124 (0.62)	12,862 (0.62)	44,037 (0.74)	57,927 (0.73)	928 (0.64)	2,740 (0.62)
Ages ≥ 70	2,981 (0.36)	4,205 (0.39)	908 (0.07)	1,746 (0.08)	5,889 (0.1)	8,948 (0.11)	348 (0.24)	1,305 (0.29)
Gender								
Males	4,638 (0.56)	6,023 (0.56)	5,953 (0.46)	9,605 (0.46)	27,567 (0.47)	37,423 (0.47)	147 (0.10)	352 (0.08)
Females	3,652 (0.44)	4,747 (0.44)	7,128 (0.54)	11,221 (0.54)	31,652 (0.53)	41,517 (0.53)	1,299 (0.90)	4,097 (0.92)
Race								
White	6,297 (0.76)	8,161 (0.76)	9,386 (0.72)	14,955 (0.72)	36,855 (0.62)	46,982 (0.60)	571 (0.39)	1,952 (0.44)
Black	1,217 (0.15)	1,506 (0.14)	1,626 (0.12)	2,219 (0.11)	9,901 (0.17)	12,393 (0.16)	819 (0.57)	2,276 (0.51)
Hispanic	575 (0.07)	817 (0.08)	1,471 (0.11)	2,590 (0.12)	8,946 (0.15)	14,275 (0.18)	41 (0.03)	154 (0.03)
Asian	201 (0.02)	286 (0.03)	598 (0.05)	1,062 (0.05)	3,517 (0.06)	5,290 (0.07)	15 (0.01)	67 (0.02)
Current Smokers	3,144 (0.38)	4,609 (0.43)	1,826 (0.14)	3,164 (0.15)	8,468 (0.14)	13,343 (0.17)	333 (0.23)	1,067 (0.24)
Myocardial Infarction	1,723 (0.21)	3,078 (0.29)	0 (0.00)	0 (0.00)	0 (0.00)	0 (0.00)	0 (0.00)	0 (0.00)
Heart Failure	1,137 (0.14)	1,580 (0.15)	187 (0.01)	253 (0.01)	2,471 (0.04)	3,106 (0.04)	53 (0.04)	153 (0.03)
Hypertension	6,667 (0.80)	8,560 (0.79)	5,165 (0.39)	7,872 (0.38)	42,625 (0.72)	55,213 (0.70)	878 (0.61)	2,829 (0.64)
Chronic Kidney Disease Stages 3–5	445 (0.05)	754 (0.07)	138 (0.01)	225 (0.01)	1,528 (0.03)	2,349 (0.03)	35 (0.02)	110 (0.02)
Liver Disease	1,968 (0.24)	2,757 (0.26)	2,918 (0.22)	4,936 (0.24)	17,185 (0.29)	23,072 (0.29)	456 (0.32)	1,346 (0.30)
Diabetes Mellitus	2,185 (0.26)	2,670 (0.25)	1,178 (0.09)	1,826 (0.09)	59,219 (1.00)	78,940 (1.00)	0 (0.00)	0 (0.00)
Diabetes with Chronic Complications	237 (0.03)	554 (0.05)	55 (0.00)	202 (0.01)	2,502 (0.04)	7,659 (0.10)	0 (0.00)	0 (0.00)
Obesity	1,668 (0.20)	2,333 (0.22)	1,650 (0.13)	3,158 (0.15)	16,736 (0.28)	25,453 (0.32)	345 (0.24)	1,132 (0.25)
Age-adjusted Charlson Score	4.00 ± 2.02	4.28 ± 2.18	1.12 ± 1.23	1.18 ± 1.30	2.21 ± 1.48	2.42 ± 1.61	1.87 ± 1.43	2.06 ± 1.39
LDL-C^b^	113.54 ± 32.95	113.79 ± 32.12	207.93 ± 20.68	207.72 ± 20.25	112.83 ± 25.93	113.33 ± 26.29	118.83 ± 26.25	118.65 ± 26.04
HDL-C^c^	50.65 ± 17.18	51.57 ± 17.60	54.98 ± 15.28	54.78 ± 15.81	51.71 ± 15.92	51.65 ± 16.42	60.48 ± 17.35	61.42 ± 18.23
Total Cholesterol	193.83 ± 37.07	196.33 ± 35.86	294.49 ± 28.25	293.44 ± 27.96	192.54 ± 31.62	193.38 ± 32.04	202.80 ± 32.20	203.51 ± 31.83
Triglycerides	142.72 ± 114.68	142.60 ± 119.93	158.59 ± 73.74	160.04 ± 73.57	140.17 ± 78.29	142.61 ± 81.51	115.85 ± 60.82	116.80 ± 69.09
Use of Other Lipid Regulating Drugs	512 (0.06)	582 (0.05)	614 (0.05)	801 (0.04)	5,144 (0.09)	5,895 (0.07)	54 (0.04)	179 (0.04)
Use of ACE-Inhibitors	3,267 (0.39)	4,092 (0.38)	1,857 (0.14)	2,746 (0.13)	18,794 (0.32)	23,697 (0.30)	374 (0.26)	1,065 (0.24)
Use of Beta Blockers	3,176 (0.38)	4,022 (0.37)	1,397 (0.11)	2,064 (0.10)	11,151 (0.19)	14,287 (0.18)	305 (0.21)	969 (0.22)
Use of Calcium Channel Blockers	2,048 (0.25)	2,698 (0.25)	1,081 (0.08)	1,782 (0.09)	10,973 (0.19)	14,341 (0.18)	299 (0.21)	986 (0.22)
Use of Diuretics	2,814 (0.34)	3,556 (0.33)	2,155 (0.16)	3,282 (0.16)	19,800 (0.33)	24,701 (0.31)	541 (0.37)	1,599 (0.36)

Patient baseline characteristics at the end of the *pseudo-immortal-time* period are summarized in either as number of patients, n (%) or mean ± standard deviation

^a^ ASCVD = Atherosclerotic Cardiovascular Disease

^b^ LDL-C = Low-density Lipoprotein Cholesterol

^c^ HDL-C = High-density Lipoprotein Cholesterol

### Statin use

Trends in statin use in 6 months increased for both the ASCVD and Diabetes groups since atorvastatin went generic in Dec 2011, with a decreasing trend for High-LDL group and a consistent trend for High-ASCVD-Risk group (Fig. 3A). 64.68% of the ASCVD group started statins within 6 months in Dec 2011, and its slope increased from + 0.6pp to + 3.15pp per year from pre- to post-guideline, reaching 76.97% statin use in May 2016 (rosuvastatin generic date). The High-LDL group started from 36.04% to 37.59% throughout the evaluation window but displayed an overall decreasing trend with a slope from -1.57pp to -0.47pp. The Diabetes group had more statin users in the post-guideline than in the pre-guideline but statin use increments per year were small (+ 0.42pp to + 0.25pp), similar to the High-ASCVD-Risk group (+ 0.78pp to -0.24pp). No significant trend changes in pre- to post-guideline trends were detected for any of the statin-benefit groups (Fig. 4).

### Optimal statin use

17,457 and 25,831 patients initiated statin treatment within 6 months following their index date in pre- and post-guideline respectively (Supplemental Table B-2), and hence, included in this analysis. Optimal (higher-intensity) statin use grew over the years and increased in slope by + 3.40pp per year on average (Fig. 3B): ASCVD group started from 32.93% in Dec 2011 and increased by + 1.89pp per year in pre-guideline, then increased slope to + 6.66pp per year in post-guideline; High-LDL group also exhibited higher slopes in post-guideline (+ 0.16pp to + 2.02pp), and the same slope improvement can be observed in Diabetes group (+ 0.70pp to + 3.08pp) and High-ASCVD-Risk group (-2.18pp and + 2.39pp). Furthermore, the changes in trend for the ASCVD group were significant (*P* < 0.001), displaying both increments in slope (long-term change) and step changes in intercept (immediate change) at guideline release. No other significant trend changes in trends were detected (Fig. 4). Note that the High-ASCVD-Risk group had high fluctuations in trend in pre-guideline as its sample size was less than 11 per month until Jan 2015.

### Statin adherence

Among statin initiators, all statin-benefit groups exhibited an increasing trend of statin adherence from Dec 2011 to May 2016 (Fig. 3C). ASCVD group adhered to statin 56.62% of the time in one year on average and statin adherence increased by + 0.18pp more per year in pre-guideline which further improved to + 2.13pp per year after guideline release. Other statin-benefit groups also had increasing trends in pre- and post-guideline. Under the Chow’s Test, no significant trend changes were detected in any statin-benefit groups (Fig. 4). We also observed high fluctuations in the post-guideline trend for the ASCVD group and in the pre-guideline trend for the High-ASCVD-Risk group.

### MACE survival

Trends of 5-Year MACE Survival were consistent over the evaluation window, except for the Diabetes group (Fig. 3D). 83.02% of the ASCVD group were MACE-free for 5 years in Dec 2011 and the slope decreased from + 1.61pp to -0.37pp in pre- to post-guideline; High-LDL group had consistently 96.25% and 95.68% of MACE-free patients in pre- and post-guideline respectively; 95.22% of Diabetes group were MACE-free for 5 years but − 0.91pp more patients did not survive MACE each year in pre-guideline, and + 0.09pp more MACE-free patients per year after guideline release; High-ASCVD-Risk group still showed great fluctuations due to low sample size in earlier months, but 92.03% of patients survived MACE on average across the evaluation period. A significant trend change was detected in the Diabetes group (*P* = 0.002), and the trajectory of 5-Year MACE Survival changed from a negative slope to a positive slope.

## Discussion

Our study aimed to evaluate the potential (long-term) impacts of the *2013 ACC/AHA Guideline* by investigating its association with significant trend changes in MACE survival and three other statin-related outcomes (statin use, optimal statin use, statin adherence) for four statin-benefit groups while controlling for the confounding effect of the availability and subsequent increase in the use of generic atorvastatin shortly before the introduction of the guideline. After separating the effect from generic atorvastatin availability, our study found that the *2013 ACC/AHA Guideline* release was associated with a statistically significant positive long-term impact (slope) on the 5-Year MACE survival for the Diabetes group (*P* = 0.002). We also found significant immediate improvements (intercept) and long-term impact (slope) in optimal statin use for the ASCVD group (*P* < 0.001) at the time of the *2013 ACC/AHA Guideline* release. Although no other significant trend changes were found (Fig. 4) after separating the effect from the generic atorvastatin availability, the four statin-related outcomes had overall positive trend changes or no further trend changes after the *2013 ACC/AHA Guideline* release.

Our findings showed that the ASCVD group had notable improvements in starting optimal (higher-intensity) statin treatment plans (as recommended in the *2013 ACC/AHA Guideline*) after the *2013 ACC/AHA Guideline* release, but no significant improvement in long-term MACE survival. On the other hand, although the Diabetes group had generally poorer health conditions at index and had no significant trend changes in statin prescription patterns (statin use and optimal statin use), the *2013 ACC/AHA Guideline* release was associated with an improvement in the downward trend of 5-Year MACE Survival significantly. It is an unexpected result because studies have found that initiating any lipid-lowering drugs in the Diabetes group can result in lower MACE occurrence [19]. As our study only followed patients’ statin usage in the first six months to understand the prescription patterns in initial consultations, we decided to follow patients’ statin use status further in a sensitivity analysis. We found that the Diabetes group was the only statin-benefit group that had increasing statin initiation in the second year after their index date (Supplemental Figure B-3). This result may indicate that although the statin initiation was only moderate in the first six months, most patients in the Diabetes group were able to start statin treatments in the second year into a higher level of LDL-C (70–189 mg/dL), which may resulted in the significant improvement that we observed in their 5-Year MACE Survival.

In our study, we found that the *2013 ACC/AHA Guideline* was still associated with increased adoption of higher-intensity statins for the ASCVD group. This finding was different from Markovitz et al. [12], but similar to Pokharel et al. and Tong et al. [14, 15]. This might be due to the flexibility in intercept term that our study allowed, as well as the other two studies [14, 15]. On the other hand, our study found no significant trend changes in statin adherence for all statin-benefit groups. This finding was similar to Yu et al. [11], also observed that the ASCVD group experienced higher statin-intensity prescriptions but no differences in statin adherence. Similarly, another study [8] further validated our finding in statin adherence, in which they showed no changes in statin adherence across all statin-benefit groups before and after the *2013 ACC/AHA Guideline* release. However, one study [10] that utilized the same data source as our study found positive association with statin adherence, but it did not consider the effect from generic atorvastatin availability.

To the authors’ knowledge, no nationally representative studies have investigated the association of the *2013 ACC/AHA Guideline* release with trend changes in MACE survival and other statin-related outcomes while controlling for the confounding effect from generic atorvastatin availability. However, this study had several limitations. (1) The study had a small sample size in the High-ASCVD-Risk group due to the lack of availability of laboratory test results in the OLDW. This might result in high variability, and hence lack of power to detect any effects in the interrupted time series analysis. (2) Under the interrupted time series analysis, all trends were assumed to be linear; this assumption would be violated, and the Chow’s test would be less powerful if, e.g., trends accelerated or plateaued non-linearly. (3) While statin use and MACE survival were evaluated on a national sample, optimal statin use and statin adherence were evaluated on statin initiators only because these outcomes can only be evaluated among patients who are taking statins. (4) We defined optimal statin use as the proportion of patients who were prescribed the same intensity statin treatment as the recommendations of the *2013 ACC/AHA Guideline* as we assumed that the guideline recommendations represent the optimal statin treatments for these defined statin-eligible groups. We recognized that “optimal” statin treatment might be different in personalized medicine and this study did not consider further individual characteristics other than those defined in the *2013 ACC/AHA Guideline*. (5) MACE survival analysis included cardiovascular death or sudden death that were only identifiable through diagnosis codes, potentially limiting event identification. However, death is a relatively rare event for our follow-up period, especially death without any prior diagnosis coding of MACE. Therefore, it is unlikely to result in significant differences in our analysis. However, the follow-up period for MACE (5 years) is longer than other statin-related outcomes in this study, which makes it more susceptible to outside influence, including the potential influence of the *2013 ACC/AHA Guideline* in the pre-guideline period as the follow-up period overlaps with the time when the *2013 ACC/AHA Guideline* was introduced. (6) This study used claims and electronic health records (EHR) data within OLDW. With that in mind, only claims or medical records available to the internal EHR system were accessible to the authors. (7) This study aimed to include only initial consultations of statin treatment as patients who have been advised of statin treatments might have different perceptions of statin treatments from patients who have not. As a result, patients were also assigned to their first statin-benefit group instead of the higher at-risk group. (8) Our result might not generalize to patients with limited access to care as only patients with at least six months of continuous enrollments before and after the index was included to ensure data completeness. (9) Health systems in the U.S. transitioned to the International Classification of Diseases, 10th Revision – ICD-10-CM in Oct 2015. We investigated the differences in coding frequencies and found higher coding frequencies in diabetes due to its granularity in ICD-10-CM. However, shortening the evaluation window to Oct 2015 did not change our conclusion (Supplemental Table B-3). (10) While our study evaluated the associations of the *2013 ACC/AHA Guideline* with statin-related outcomes that may indicate the guideline’s potential impact, this study cannot infer the causal effect of the guideline. Observed changes in outcome trends could be partially attributable to changes in the underlying risk profile of the study population; how to disentangle such systematic changes from the effects of the guidelines and generic statin availability requires further investigation.

## Conclusions

Our study found that the release of the *2013 ACC/AHA Guideline* was associated with significant positive potential impacts on a few statin-related outcomes after separating the effect from the generic atorvastatin availability. Specifically, we observed significant trend improvement in 5-Year MACE Survival for the Diabetes group after the *2013 ACC/AHA Guideline* release. Although immediate improvement in statin usage was not observed, the Diabetes group experienced increasing statin use in the second year, which may explain the observed significant reduction of the occurrence of MACE in the long term. We also observed immediate improvements in optimal (higher-intensity) statin use for the ASCVD group after the *2013 ACC/AHA Guideline* release. These significant associations might suggest that [1] the potential impact of the *2013 ACC/AHA Guideline* on statin use may be delayed but may be observed in the long run, and [2] healthcare professionals may be more responsive to the guidelines and more aggressive in statin prescriptions for higher at-risk groups. While these significant associations may indicate the potential impacts that the *2013 ACC/AHA Guideline* might have, further investigation is required to infer the causal relationship between *2013 ACC/AHA Guideline* and statin-related outcomes.

### Electronic supplementary material

Below is the link to the electronic supplementary material.

Supplementary Material 1

## Data Availability

The data that support the findings of this study are available from Optum Labs but restrictions apply to the availability of these data, which were used under license for the current study, and so are not publicly available. Data are however available from the authors upon reasonable request and with permission of Optum Labs.

## Abbreviations

2013 ACC/AHA Guideline: 2013 ACC/AHA Guideline on the Treatment of Blood Cholesterol to Reduce Atherosclerotic Cardiovascular Risk in Adults
ACC: American College of Cardiology
AHA: American Heart Association
ASCVD: Atherosclerotic Cardiovascular Disease
EHR: Electronic health records
FDA: Food and Drug Administration
ICD: International Classification of Diseases
LDL-C: Low-density Lipoprotein Cholesterol
MACE: Major Adverse Cardiac Events
OLDW: Optum Labs Database Warehouse
U.S.: United States
