# Supplementary material for: Potential impact of blood cholesterol guidelines on statin treatment in the U.S. population using interrupted time series analysis
Source: BMC Cardiovasc Disord. 2024 May 10;24:245. doi: 10.1186/s12872-024-03921-z (PMC11088177; doi:10.1186/s12872-024-03921-z)
Supplement: Supplementary file 1 — Supplementary Material 1 [file 12872_2024_3921_MOESM1_ESM.docx]

# **Supplemental Material**

Table of Contents

[Supplemental Material 1](#_Toc157712850)

[A. Supplemental Methods 4](#_Toc157712851)

[B. Supplemental Results 9](#_Toc157712852)

Table of Supplemental Tables

[Supplemental Table A‑1: Medical Coding to Condition Mapping. 5](#_Toc157712853)

[Supplemental Table B‑1: Patient Baseline Characteristics at Pseudo-immortal-time Period. 12](#_Toc157712854)

[Supplemental Table B‑2: Statin-Related Outcomes 15](#_Toc157712855)

[Supplemental Table B‑3: The Chow’s Test P-values Adjusted for the ICD-10-CM Transition. 19](#_Toc157712856)

Table of Supplemental Figures

[Supplemental Figure A‑1: The *2013 ACC/AHA Guideline* Recommendations. 8](#_Toc157712857)

[Supplemental Figure B‑1: Historical Distribution of Statin Usage Across U.S. Regions. 10](#_Toc157712858)

[Supplemental Figure B‑2: Historical Distribution of Simvastatin and Atorvastatin Usage. 11](#_Toc157712859)

[Supplemental Figure B‑3: Year 2 Statin Initiation Per 1000. 17](#_Toc157712860)

## **Supplemental Methods**

To identify the four statin-benefit groups defined by the *2013* *ACC/AHA Guideline*, the following definitions were used:

1. ASCVD: Patients who were aged 21 years old and above with clinical ASCVD between Dec 2011 and May 2016, including initial encounters of acute myocardial infarction, angina with coronary artery disease, ischemic stroke, transient ischemic attack, or revascularization procedures during hospitalizations or emergency room visits. Excluded patients with historical ASCVD.
2. High-LDL: Patients who were aged 21 years old and above with LDL-C levels greater or equal to 190 mg/dL between Dec 2011 and May 2016. Excluded patients with historical ASCVD.
3. Diabetes: Patients who were aged 40-75 years old with LDL-C levels 70-189 mg/dL between Dec 2011 and May 2016, and have had a diagnosis of diabetes at any time prior to the LDL-C result. Excluded patients with historical ASCVD.
4. High-ASCVD: Patients aged 40-75 years old with 10-year ASCVD risk $\geq$ 7.5% with LDL-C levels 70-189 mg/dL between Dec 2011 and May 2016. Excluded patients with historical ASCVD or diagnosis of diabetes prior.

Note that this study focuses on the initial consultation of statin treatment. As a result, patients who met any of the criteria above before the start of the evaluation period, Dec 2011, were excluded.

**Supplemental Table A‑1: Medical Coding to Condition Mapping.**

| Conditions/Procedures | Coding |
| --- | --- |
| Myocardial Infarction (Initial Encounter) | ICD-9-CM: 410.x1  ICD-10-CM: I21.x |
| Myocardial Infarction (Subsequent Encounters) | ICD-9-CM: 410.x (exclude 410.x1)  ICD-10-CM: I22.x |
| History of Myocardial Infarction | ICD-9-CM: 411.0, 412  ICD-10-CM: I23, I24.1, I25.2 |
| Angina | ICD-9-CM: 413.x  ICD-10-CM: I20.x, I23.7, I25.11x, I25.7x |
| Ischemic Stroke (Initial Encounter) | ICD-9-CM: 433.x1, 434.x1, 436.x  ICD-10-CM: I63.x |
| Ischemic Stroke (Subsequent Encounters) | ICD-10-CM: I69.3x |
| Transient Ischemic Attack | ICD-9-CM: 435.x  ICD-10-CM: G45.x |
| Coronary Artery Disease | ICD-9-CM: 411.x, 414.x (exclude 414.10, 414.19)  ICD-10-CM: I24.x, I25.x (exclude I25.3) |
| Cardiovascular Death | ICD-9-CM: 427.5, V12.53  ICD-10-CM: I46.1, I46.9 |
| Diabetes Mellitus | ICD-9-CM: 250.x, 357.2, 362.0x, 366.41  ICD-10-CM: E08.x-E14.x |
| History of Percutaneous Coronary Intervention | ICD-9-CM: V45.82  ICD-10-CM: Z98.61, Z95.5 |
| History of Coronary Artery Bypass Grafts | ICD-9-CM: V45.81  ICD-10-CM: Z95.1 |
| Percutaneous Coronary Intervention | CPT-4: 92920, 92921, 92924, 92925, 92928, 92929, 92933, 92934, 92937, 92938, 92941, 92943, 92944, 92975, 92977, 92980, 92981, 92982, 92984, 92995, 92996  ICD-9-PCS: 00.66, 36.0x  ICD-10-PCS: 0270xxx-0273xxx, 02C0xxx, 02C1xxx, 02C2xxx, 02C3xxx |
| Coronary Artery Bypass Grafts | CPT-4: 33510, 33511, 33512, 33513, 33514, 35515, 33516, 33517, 33518, 33519, 33521, 33522, 33523, 33530, 33533, 33534, 33535, 33536, 4110F  ICD-9-PCS: 36.1x  ICD-10-PCS: 02100xx, 02110xx, 02120xx, 02130xx |
| Cancer | ICD-9-CM: 140.x-165.x, 170.x-175.x, 179.x- 195.x  ICD-10-CM: C00.x-C26.x, C30.x-C34.x, C37.x-C41.x, C43.x-C58.x, C4A.x, C60.x-C75.x, C7A.x, C81.x-C86.x, C88.x, C90.x-C96.x |
| Myopathy | ICD-9-CM: 359.4, 359.9, 359.81, 359.89, 729.1, 728.81  ICD-10-CM: G72.x, G73.7, M60.1, M60.8, M60.9 |
| Rhabdomyolysis | ICD-9-CM: 728.88  ICD-10-CM: M62.82 |
| Hospice Care/Skilled Nursing Facilities | Place of Service Code: 31, 34 |
| Sudden Death | ICD-9-CM: 798(.x)  ICD-10-CM: R99 |


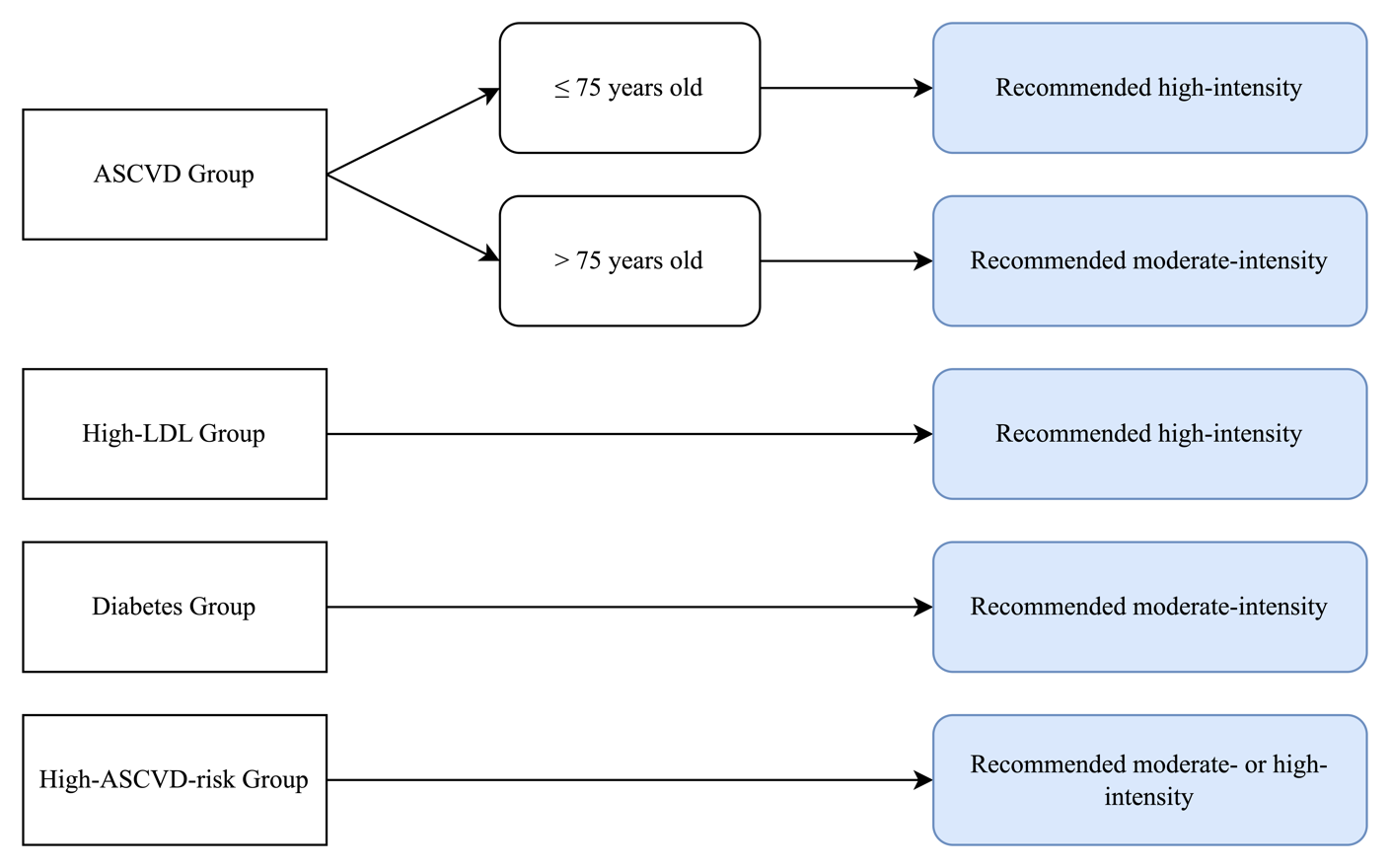


**Supplemental Figure A‑1: The *2013 ACC/AHA Guideline* Recommendations.**

The four white boxes on the left represented the four statin-benefit groups identified by the *2013 ACC/AHA Guideline*. The arrows showed the *2013 ACC/AHA Guideline* recommendation flowcharts to the Class 1 recommendations of statin intensity. The blue boxes represented the final recommendation for statin treatment intensity.

## **Supplemental Results**


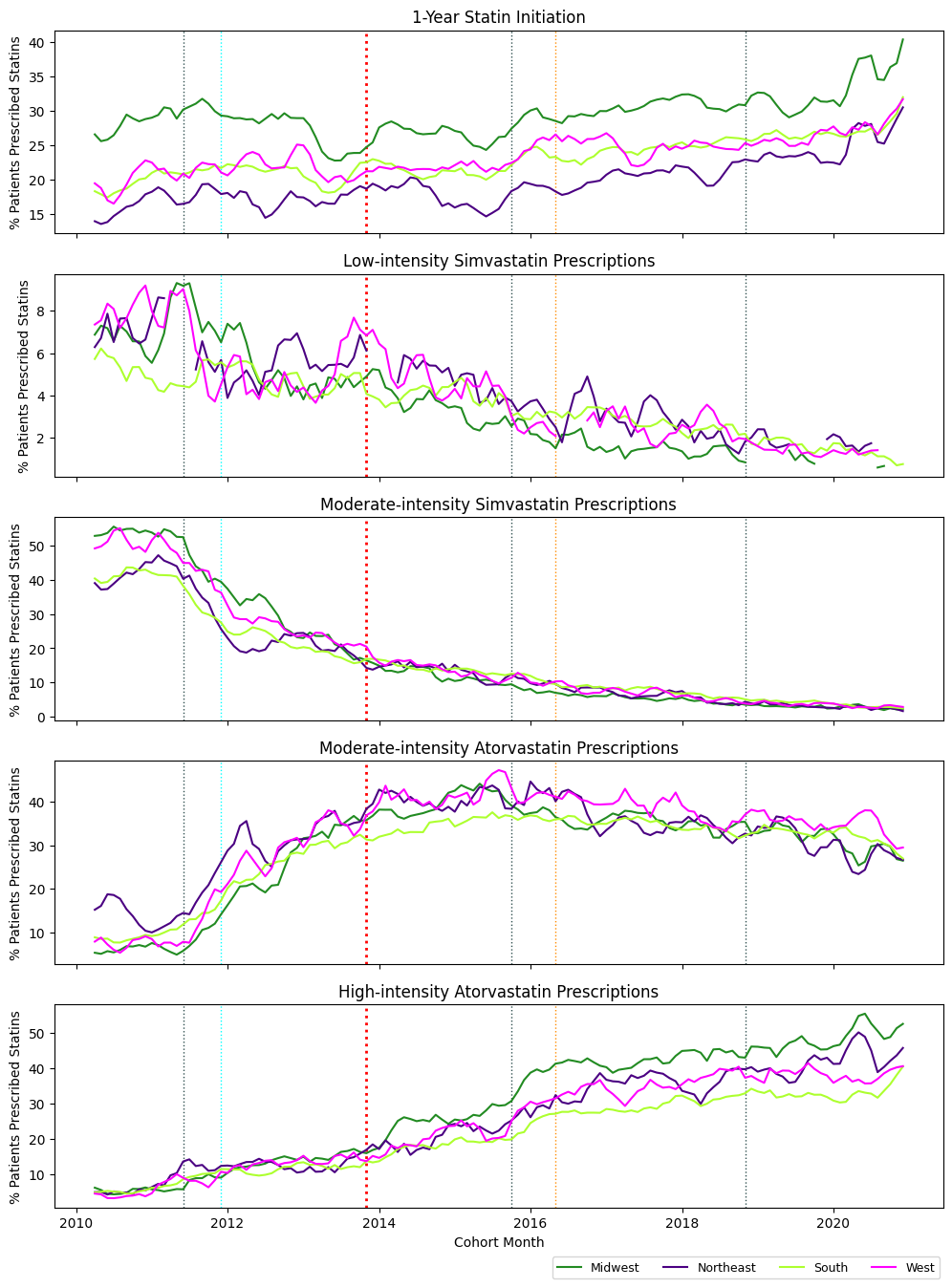


**Supplemental Figure B‑1: Historical Distribution of Statin Usage Across U.S. Regions.**

The figure above shows statin prescription distribution across four major U.S. regions (Midwest, Northeast, South, and West) from 2010-2020 using a 4-month moving average. There were 6 vertical dotted lines over the year representing major statin and healthcare-related events in the U.S.: the restriction on simvastatin 80mg in Jun 2011, atorvastatin became generic in Dec 2011, the release of the 2013 ACC/AHA Guideline in Nov 2013, ICD-10-CM Transition in Oct 2015, rosuvastatin became generic in May 2016, and the release of the 2018 guideline in Nov 2018.

***
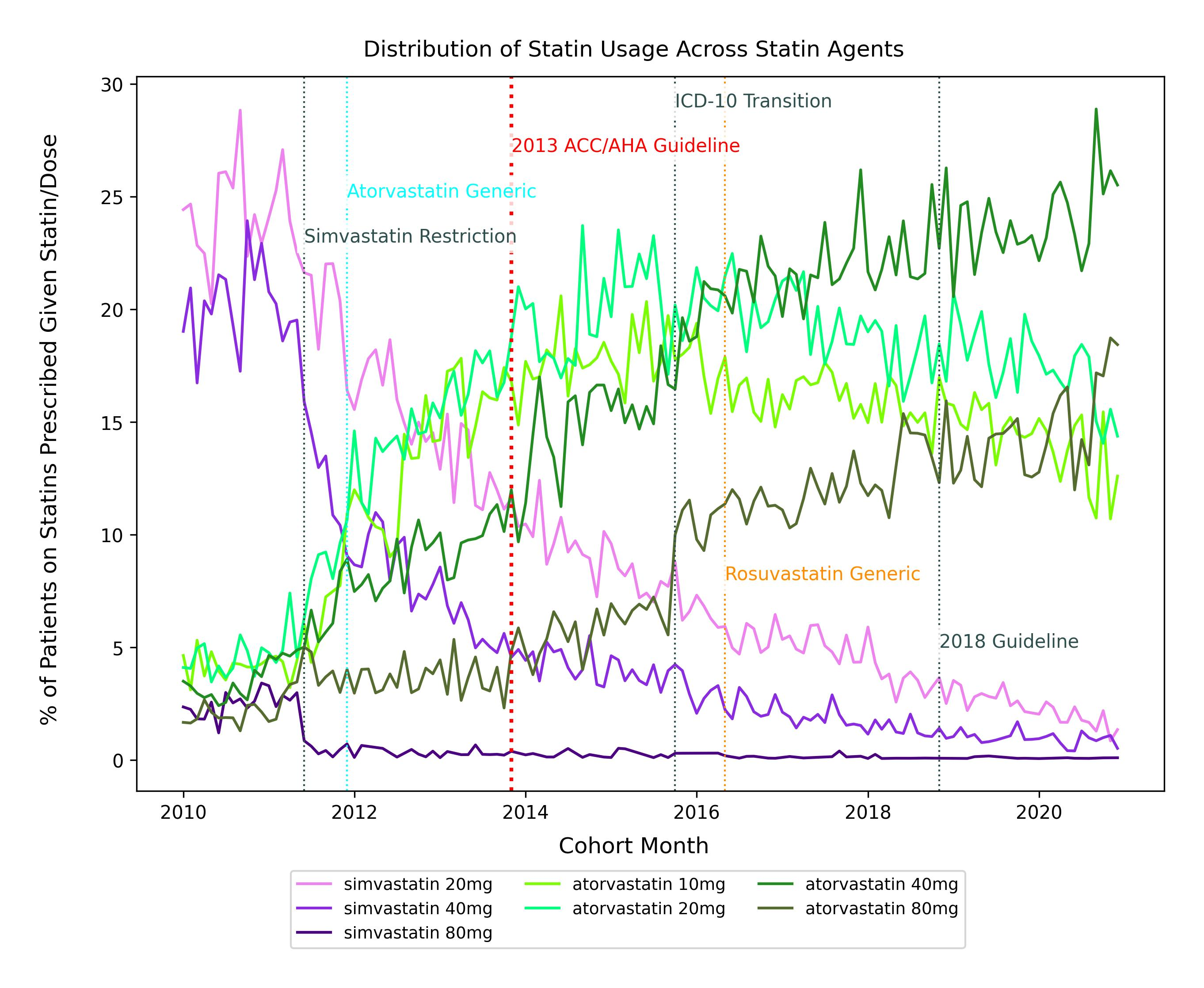
***

**Supplemental Figure B‑2: Historical Distribution of Simvastatin and Atorvastatin Usage.**

The figure shows statin prescription distribution across different dosages of simvastatin and atorvastatin from 2010-2020 using a 4-month moving average. There were 6 vertical dotted lines over the year representing major statin and healthcare-related events in the U.S.: the restriction on simvastatin 80mg in Jun 2011, atorvastatin became generic in Dec 2011, the release of the 2013 ACC/AHA Guideline in Nov 2013, ICD-10-CM Transition in Oct 2015, rosuvastatin became generic in May 2016, and the release of the 2018 guideline in Nov 2018.

**Supplemental Table B‑1: Patient Baseline Characteristics at Pseudo-immortal-time Period.**

|  | ASCVD^a^ | | High-LDL | | Diabetes | | High-ASCVD-Risk | |
| --- | --- | --- | --- | --- | --- | --- | --- | --- |
|  | Pre-Guideline (n=8,290) | Post-Guideline (n=10,770) | Pre-Guideline (n=13,081) | Post-Guideline (n=20,826) | Pre-Guideline (n=59,219) | Post-Guideline (n=78,940) | Pre-Guideline (n=1,446) | Post-Guideline (n=4,449) |
| Age | 63.57 ± 12.71 | 64.54 ± 13.41 | 51.42 ± 12.48 | 51.91 ± 12.85 | 56.11 ± 9.73 | 56.64 ± 9.95 | 59.97 ± 10.80 | 61.77 ± 10.58 |
| Females | 3,652 (0.44) | 4,747 (0.44) | 7,128 (0.54) | 11,221 (0.54) | 31,652 (0.53) | 41,517 (0.53) | 1,299 (0.90) | 4,097 (0.92) |
| Race | | | | | | | | |
| White | 6,297 (0.76) | 8,161 (0.76) | 9,386 (0.72) | 14,955 (0.72) | 36,855 (0.62) | 46,982 (0.60) | 571 (0.39) | 1,952 (0.44) |
| Black | 1,217 (0.15) | 1,506 (0.14) | 1,626 (0.12) | 2,219 (0.11) | 9,901 (0.17) | 12,393 (0.16) | 819 (0.57) | 2,276 (0.51) |
| Hispanic | 575 (0.07) | 817 (0.08) | 1,471 (0.11) | 2,590 (0.12) | 8,946 (0.15) | 14,275 (0.18) | 41 (0.03) | 154 (0.03) |
| Asian | 201 (0.02) | 286 (0.03) | 598 (0.05) | 1,062 (0.05) | 3,517 (0.06) | 5,290 (0.07) | 15 (0.01) | 67 (0.02) |
| Current Smokers | 3,322 (0.40) | 4,909 (0.46) | 2,090 (0.16) | 3,633 (0.17) | 9,548 (0.16) | 15,337 (0.19) | 357 (0.25) | 1,182 (0.27) |
| Myocardial Infarction | 1,724 (0.21) | 3,081 (0.29) | 0 (0.00) | 0 (0.00) | 0 (0.00) | 0 (0.00) | 0 (0.00) | 0 (0.00) |
| Hypertension | 7,117 (0.86) | 9,148 (0.85) | 5,662 (0.43) | 8,670 (0.42) | 44,235 (0.75) | 57,526 (0.73) | 921 (0.64) | 2,924 (0.66) |
| Diabetes Mellitus | 2,417 (0.29) | 2,974 (0.28) | 1,513 (0.12) | 2,413 (0.12) | 59,219 (1.00) | 78,940 (1.00) | 65 (0.04) | 249 (0.06) |
| Diabetes with Chronic Complications | 325 (0.04) | 787 (0.07) | 81 (0.01) | 414 (0.02) | 3,161 (0.05) | 11,421 (0.14) | <11 (0.00) | 26 (0.01) |
| Obesity | 1,859 (0.22) | 2,693 (0.25) | 2,037 (0.16) | 3,857 (0.19) | 18,616 (0.31) | 28,485 (0.36) | 384 (0.27) | 1,265 (0.28) |
| Age-adjusted Charlson Score | 4.18 ± 2.19 | 4.53 ± 2.37 | 1.25 ± 1.38 | 1.33 ± 1.47 | 2.29 ± 1.62 | 2.56 ± 1.81 | 2.02 ± 1.65 | 2.23 ± 1.60 |
| LDL-C^b^ | 84.36 ± 32.35 | 78.87 ± 32.87 | 143.33 ± 44.14 | 143.19 ± 45.51 | 105.98 ± 29.00 | 105.40 ± 29.65 | 112.90 ± 29.98 | 114.09 ± 29.35 |
| HDL-C^c^ | 49.10 ± 15.68 | 49.76 ± 16.15 | 53.88 ± 15.06 | 53.72 ± 15.74 | 49.56 ± 15.20 | 49.57 ± 15.62 | 58.24 ± 18.03 | 58.63 ± 16.24 |
| Use of Other Lipid Regulating Drugs | 669 (0.08) | 693 (0.06) | 897 (0.07) | 1,156 (0.06) | 5,799 (0.10) | 6,605 (0.08) | 65 (0.04) | 193 (0.04) |
| Use of ACE Inhibitors | 4,071 (0.49) | 5,098 (0.47) | 2,251 (0.17) | 3,384 (0.16) | 20,941 (0.35) | 26,740 (0.34) | 398 (0.28) | 1,144 (0.26) |
| Use of Beta Blockers | 3,840 (0.46) | 4,893 (0.45) | 1,617 (0.12) | 2,381 (0.11) | 12,144 (0.21) | 15,631 (0.20) | 342 (0.24) | 1,038 (0.23) |

Patient baseline characteristics at index are summarized in either as number of patients, n (%) or mean ± standard deviation.

^a^ ASCVD = Atherosclerotic Cardiovascular Disease

^b^ HDL-C = High-density Lipoprotein Cholesterol

^c^ LDL-C = Low-density Lipoprotein Cholesterol

**Supplemental Table B‑2: Statin-Related Outcomes**

| *2013 ACC/AHA Guideline^a^* Statin-Benefit Group | | | | | | | | |
| --- | --- | --- | --- | --- | --- | --- | --- | --- |
|  | **ASCVD**^b^ | | **High-LDL^c^** | | **Diabetes** | | **High-ASCVD-Risk** | |
|  | Pre-Guideline | Post-Guideline | Pre-Guideline | Post-Guideline | Pre-Guideline | Post-Guideline | Pre-Guideline | Post-Guideline |
| **Statin Use** | | | | | | | | |
| Sample Size | 8,290 | 10,770 | 13,081 | 20,826 | 59,219 | 78,940 | 1,446 | 4,449 |
| Weighted Average | 66.27 % | 71.77 % | 38.20 % | 36.38 % | 11.64 % | 13.04 % | 5.33 % | 5.24 % |
| Slope per Year | +0.61 pp | +3.15 pp | -1.57 pp | -0.47 pp | +0.42 pp | +0.25 pp | +0.78 pp | -0.24 pp |
| Chow’s P-value | - | 0.2069 | - | 0.5821 | - | 0.4638 | - | 0.5147 |
| **Guideline Adherence** | | | | | | | | |
| Sample Size | 5,494 | 7,730 | 4,996 | 7,577 | 6,890 | 10,291 | 77 | 233 |
| Weighted Average | 35.11 % | 53.36 % | 15.69 % | 21.30 % | 74.69 % | 80.01 % | 72.73 % | 81.55 % |
| Slope per Year | +1.89 pp | +6.66 pp | +0.16 pp | +2.02 pp | +0.70 pp | +3.08 pp | -2.18 pp | +2.39 pp |
| Chow’s P-value | - | 0.0002 | - | 0.0903 | - | 0.0670 | - | 0.9827 |
| **Statin Adherence** | | | | | | | | |
| Sample Size | 5,494 | 7,730 | 4,996 | 7,577 | 6,890 | 10,291 | 77 | 233 |
| Weighted Average | 58.44 % | 61.41 % | 47.12 % | 49.85 % | 51.61 % | 52.30 % | 51.41 % | 52.04 % |
| Slope per Year | +0.18 pp | +2.13 pp | +1.32 pp | +0.42 pp | +0.58 pp | +0.67 pp | +17.21 pp | +3.84 pp |
| Chow’s P-value | - | 0.3355 | - | 0.5204 | - | 0.8296 | - | 0.4180 |
| **MACE^d^ Survival (5-Year)** | | | | | | | | |
| Sample Size | 8,290 | 10,770 | 13,081 | 20,826 | 59,219 | 78,940 | 1,446 | 4,449 |
| Weighted Average | 85.75 % | 85.62 % | 96.25 % | 95.68 % | 95.37 % | 95.33 % | 90.73 % | 92.46 % |
| Slope per Year | +1.61 pp | -0.37 pp | -0.42 pp | -0.37 pp | -0.91 pp | +0.09 pp | +1.49 pp | -3.56 pp |
| Chow’s P-value | - | 0.2741 | - | 0.9090 | - | 0.0023 | - | 0.5754 |

^a^ *2013 ACC/AHA Guideline* = 2013 American College of Cardiology/American Heart Association Guideline on the Treatment of Blood Cholesterol to Reduce Atherosclerotic Cardiovascular Risk in Adults

^b^ ASCVD = Atherosclerotic Cardiovascular Disease

^c^ LDL = Low-density Lipoprotein

^d^ MACE = Major Adverse Cardiac Event


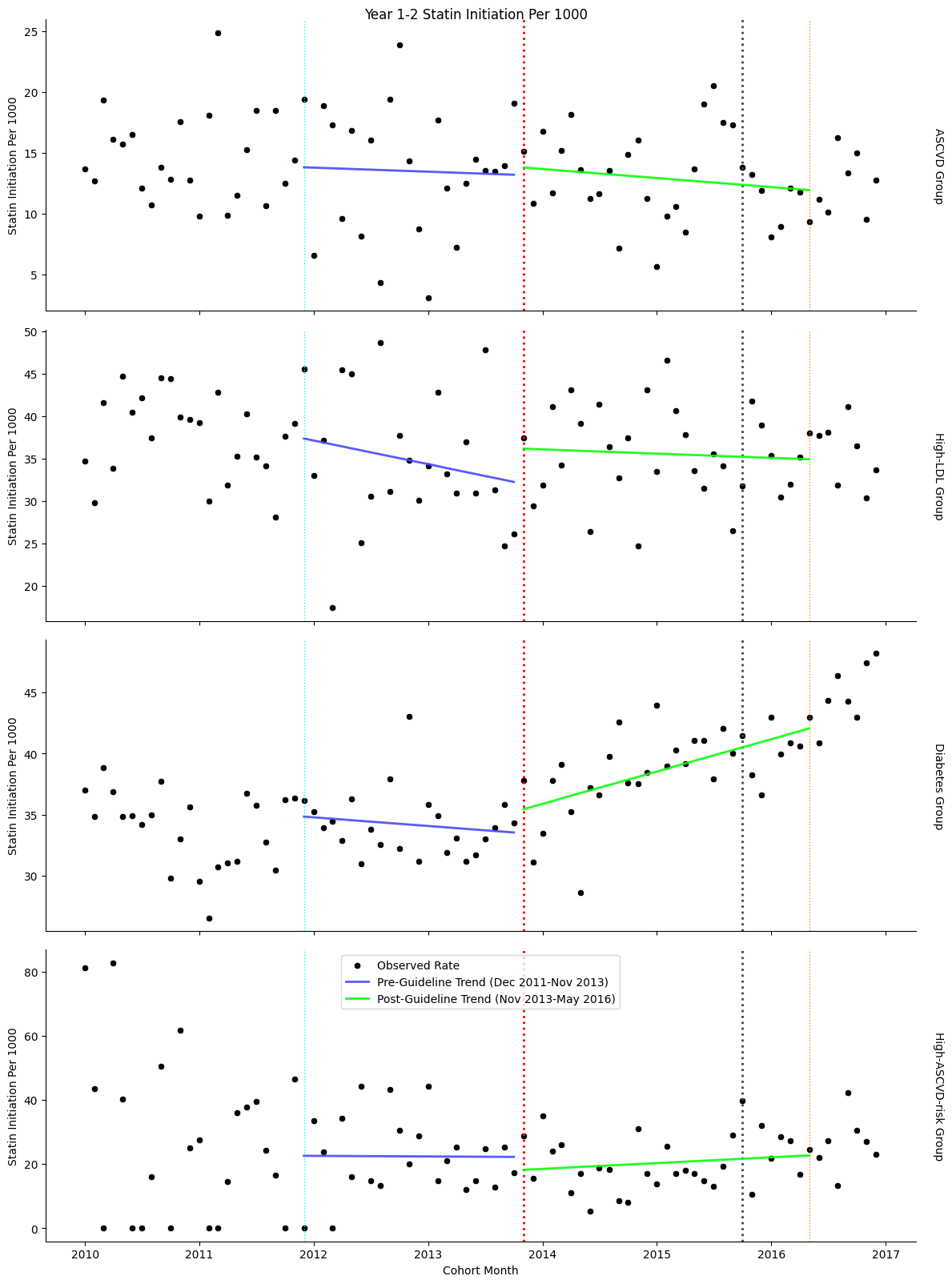


**Supplemental Figure B‑3: Year 2 Statin Initiation Per 1000.**

The figure above showed statin initiation in the second year per 1000 person-years. There were 4 vertical dotted lines representing major statin and healthcare-related events in the U.S.: atorvastatin became generic in Dec 2011, the release of the 2013 guideline in Nov 2013, ICD-10-CM Transition in Oct 2015, and rosuvastatin became generic in May 2016.

**Supplemental Table B‑3: The Chow’s Test P-values Adjusted for the ICD-10-CM Transition.**

|  | ASCVD^a^ | High-LDL^b^ | Diabetes | High-ASCVD-Risk |
| --- | --- | --- | --- | --- |
| Statin Use | 0.597 | 0.800 | 0.389 | 0.627 |
| Guideline Adherence | <0.001 | 0.025 | 0.111 | 0.991 |
| Statin Adherence | 0.490 | 0.363 | 0.750 | 0.500 |
| 5-Year MACE^c^ | 0.342 | 0.600 | 0.009 | 0.567 |

The table above showed the p-values from Chow’s test when the evaluation window was shifted to avoid coding differences from the ICD-10-CM Transition. The evaluation window was adjusted from atorvastatin generic date in Dec 2011 to ICD-10-CM Transition in Oct 2015.

^a^ ASCVD = Atherosclerotic Cardiovascular Disease

^b^ LDL = Low-density Lipoprotein

^c^ MACE = Major Adverse Cardiac Events
